# The Positive Relationship Between the Low-Density Lipoprotein Cholesterol/Apoprotein B Ratio and Bone Turnover Markers in Patients With Type 2 Diabetes

**DOI:** 10.3389/fendo.2022.903336

**Published:** 2022-06-09

**Authors:** Chun-feng Lu, Wang-shu Liu, Hai-yan Huang, Xiao-qin Ge, Ling-yan Hua, Xue-qin Wang, Jian-bin Su

**Affiliations:** ^1^ Department of Endocrinology, Affiliated Hospital 2 of Nantong University and First People’s Hospital of Nantong City, Nantong, China; ^2^ Department of Ophthalmology, Affiliated Hospital 2 of Nantong University and First People’s Hospital of Nantong City, Nantong, China

**Keywords:** type 2 diabetes, bone turnover, low-density lipoprotein cholesterol/apolipoprotein B ratio, small and density low-density lipoprotein cholesterol, osteocalcin, C-terminal telopeptide, N-terminal propeptide of type-I procollagen

## Abstract

**Background:**

Dyslipidemia may contribute to low bone turnover in patients with type 2 diabetes (T2D) through mediating oxidative stress and atherosclerosis. The low-density lipoprotein cholesterol/apoprotein B (LDL-C/Apo B) ratio is a surrogate marker of small and density low-density lipoprotein cholesterol (sd-LDL-C), a most harmful group of LDL-Cs. The present study aimed to investigate the association between the LDL-C/Apo B ratio and bone turnover in patients with T2D.

**Methods:**

This study was a cross-sectional study enrolled patients with T2D from January 2021 to December 2021. Each participant was assessed for lipid profiles, bone turnover markers (BTMs), lumbar spine (L1-L4) and hip dual-energy X-ray absorptiometry (DXA) scans. Osteoporosis was diagnosed as a T-score lower than or equal to -2.5 at the spine or hip.

**Results:**

A total of 335 patients with T2D were enrolled in the study, and the LDL-C/Apo B ratio ranged from 0.78 to 4.00. Along with the LDL-C/Apo B ratio tertile ascending, osteocalcin (OC), C-terminal telopeptide (CTx) and N-terminal propeptide of type-I procollagen (PINP) levels gradually increased (all *p <* 0.05). There were no differences in lumbar spine and hip T-score, proportion of osteoporosis (all p > 0.05) among the three subgroups. The LDL-C/Apo B ratio was positively correlated with lnOC (*r =* 0.244, *p <* 0.001), lnCTx (*r =* 0.226, *p <* 0.01) and lnPINP (*r =* 0.211, *p <* 0.001). These significant positive correlations persisted even when divided into male and female subgroups. Furthermore, three multiple linear regression analyses were constructed to investigate the independent association of the LDL-C/Apo B ratio with the BTMs levels. After adjusting for other clinical parameters, the LDL-C/Apo B ratio was still significantly associated with OC level (*β* = 0.199, *t* = 3.348, *p* < 0.01), CTx level (*β* = 0.238, *t* = 4.084, *p* < 0.001) and PINP level (*β* = 0.162, *t* = 2.741, *p* < 0.01).

**Conclusion:**

The LDL-C/Apo B ratio was significantly and positively associated with BTMs in patients with T2D. In clinical practice, more attention should be paid to the patients with T2D whose LDL-C/Apo B ratio is relatively low for the purpose of maintaining bone health.

## Introduction

Type 2 diabetes (T2D) and its chronic complications have posed a great threat to global public health, among which bone fragility has attracted more and more attention due to its high incidence, high disability rate and serious impact on quality of life (1). Bone fragility may contribute to an up to three-fold increased risk of lip fractures and more common wrist and foot fractures in people with T2D than in healthy people (2). However, bone mineral density (BMD) is not sufficient to reflect alternations in bone fragility in patients with T2D, as studies have shown that these patients have a 5-10% increase in BMD compared to their peers without T2D (3). The maintenance of bone health requires the continuous replacement of worn bone tissue with newly synthesized calcified bone matrix throughout life, a process named as bone turnover (4). Bone turnover markers (BTMs) include osteocalcin (OC), C-terminal telopeptide (CTx) and N-terminal propeptide of type-I procollagen (PINP), and a series of studies have consistently concluded that BTMs were significantly reduced and were associated with increased risks of fragility fracture in patients with T2D compared to healthy controls (5). Therefore, it is of great significance to discover and timely intervene the risk factors of low bone turnover in patients with T2D to improve the prognosis of these patients.

T2D is usually accompanied by hyperlipidemia due to insulin resistance, and hyperlipidemia may be involved in multiple chronic complications of T2D (6). *In vitro* studies showed that oxidized lipids reduced bone turnover by inhibiting osteoblast differentiation and inducing osteoclast differentiation (7). Low-density lipoprotein cholesterol (LDL-C) is a heterogeneous group of lipoproteins, among which with smaller sizes and heavier densities were known as small and density LDL-C (sdLDL-C) (8). Compared with other LDL-Cs, sdLDL-C is characterized by low affinity with LDL receptor, long half-life, susceptible to oxidation, easy to penetrate into the artery wall and so on (9). The LDL-C/apolipoprotein B (LDL-C/Apo B) ratio is a surrogate marker of LDL particle size, and the smaller the LDL-C/Apo B ratio is, the more dominant SD-LDL particles are in LDL particles (10). Due to the methods of detecting sd-LDL-C are laborious, the LDL-C/Apo B ratio is commonly used in clinical work and scientific research (10). Clinical studies revealed that the LDL-C/Apo B ratio was significantly negatively associated with instability of atherosclerotic coronary atherosclerotic plaques (11) and restenosis of coronary stents (12). As bone is highly vascularized connective tissue, affecting the blood supply for bone tissue contributes to the decline of bone turnover (13). The LDL-C/Apo B ratio was closely related to atherosclerosis and oxidative stress, which both were major pathogenesis of low bone turnover in patients with T2D. Therefore, we speculated that the LDL-C/Apo B ratio might be closely associated with BTMs in patients with T2D; those with a lower LDL-C/Apo B ratio may have greater suppression in bone turnover than those with a higher LDL-C/Apo B ratio. However, few studies have explored the relationship between the two.

In the present study, we aimed to estimate the relationship of the LDL-C/Apo B ratio with BTMs in patients with T2D.

## Methods

### Study Design and Participants

This was an observational cross-sectional study which was performed among patients diagnosed with T2D at the Second Affiliated Hospital of Nantong University between January 2021 to October 2021 (14). The main exclusion criteria were as follows: type 1 diabetes, previous use of steroids and anabolic steroids, previous use of antiosteoporosis drugs (e.g., vitamin D, calcium tablet, bisphosphonates, denosumab and selective estrogen receptor modulators), previous or current received antiandrogen therapy, previous use of proprotein convertase subtilisin/kexin type 9 (PCSK9) inhibitors, previous or current malignant tumors, chronic hepatitis and heart failure, acute diabetic complications, history of lumbar surgery, history of thyroid or parathyroid disease. Finally, total 335 patients with T2D were included in the present study. Written informed consent was given after each subject fully understood the present study protocol. The study followed the Declaration of Helsinki thoroughly and was approved by the medical research ethics committee of the Second Affiliated Hospital of Nantong University.

### Basic Data Collection

Clinical data including the demographic data, lifestyle, medication history and diagnosis history of diseases were collected by interviewing and examining each participant upon enrollment. Body mass index (BMI) was calculated as the weight/height squared. Blood pressure was measured by a standard mercury sphygmomanometer, and the average of three recordings was recorded.

### Laboratory Examination

Fasting blood samples and fresh first-void morning urine samples were collected for respective measurement of blood indexes, urinary albumin and urinary creatinine after enrollment. The urinary albumin-to-creatinine ratio (UACR) was calculated as the ratio of urinary albumin to urinary creatinine. Lipid profiles such as triglycerides (TG), total cholesterol (TC), high-density lipoprotein (HDL-C), LDL-C, apoprotein A (Apo A) and apoprotein B (Apo B) levels, glucose, uric acid (UA), creatinine (Cr) and cystatin C levels were measured using an automated biochemical analyzer (Model 7600, Hitachi) with the inter-assay and intra-assay coefficients of variation (CVs) < 2.8%, and the LDL-C/Apo B ratio was calculated as the ratio of LDL-C to Apo B ratio. According to the CKD-EPI creatinine-cystatin C equation, estimated glomerular filtration rate (eGFR) was calculated (2012) (15). Chronic kidney disease (CKD) was defined based on an eGFR < 60 ml/min/1.73 m^2^ or a UACR ≥ 30 mg/g lasting more than 3 months (16). HbA1c levels were measured with an ion exchange-based high-performance liquid chromatography (HPLC) method in a hemoglobin analysis system (D-10, Bio-Rad) with the inter-assay and intra-assay CVs < 3.0%. Serum C-peptide (CP) levels were measured by the chemiluminescence method in an immunoassay system (DxI 800, Beckman Coulter) with the inter-assay and intra-assay CVs < 4.0%. In order to eliminate the influence of exogenous insulin, HOMA-IR_CP_ which was defined as (fasting glucose × fasting CP)/22.5 was adopted as an indicator of insulin resistance (17). Serum OC, CTx, PINP, parathyroid hormone (PTH) and vitamin D levels were analyzed on an automated immunoassay system (iSYS, Immunodiagnostic Systems Ltd., Boldon) using a chemiluminescence method.

The corresponding CVs from the manufacturers as follows: OC < 2.0%, CTx < 5.4%, PINP < 4.5%, PTH < 4.5%, and vitamin D < 4.0%.

### Diagnosis of Peripheral Artery Disease

The ankle-brachial index (ABI) was detected in each participant using the color Doppler blood flow device (Chioy Medical, Beijing) under the operation of an experienced physician. Then peripheral artery disease (PAD) was diagnosed with reference to the Inter-Society Consensus for the Management of Peripheral Arterial Disease guideline and based the ABI values (18).

### Measurement of Bone Mineral Density

All participants underwent spine (L1-L4) and hip dual-energy X-ray absorptiometry (DXA) scans on Prodigy Scanners (GE-Healthcare, Madison) by trained investigators, and the results were analyzed according to the manufacturer’s recommendations. The calculation of T-score was based on the peak BMD in healthy young people of the same race and gender, and the calculation formula of T-score was (measured BMD - peak BMD in healthy young people of the same race and gender)/standard deviation of peak BMD in healthy young people of the same race and sex. The daily CV value of DXA was controlled below 0.24%. Average T-scores of L1-L4 and T-scores of hips were recorded for further analyses. Osteoporosis was diagnosed as a T-score lower than or equal to -2.5 at the spine or hip (19).

### Statistical Analyses

The total participants were divided into three subgroups based on the LDL-C/Apo B ratio. Kolmogorov-Smirnov test was first conducted to test whether continuous variables conformed to normal distribution. In order to achieve a normal distribution for further analysis, a natural logarithm transformation (ln) was applied, such as lnOC, lnCTX and lnPNIP. The normally and skewed distributed continuous variables and the categorical variables were respectively described as mean ± SD, median (25 and 75% interquartile) and frequencies (percentages). We adopted the one-way analysis of variance, the Kruskal-Wallis test and the chi-square test to compare differences in normally distributed data, skewed distributed data and categorical data, respectively. Pearson’s bivariate correlation analyses were applied to investigate the correlations of the LDL-C/Apo B ratio with BTMs in the total population and separately in men and women. Furthermore, we constructed three multiple linear regression analyses to investigate the independent association of the LDL-C/Apo B ratio with the BTMs levels. Before conducting the linear regression analyses, the case analyses screening outliers were carried out first. If the standardized residual values were not in the range of -3 to 3, the outliers should be considered. Later, the case records were retrieved to exclude the abnormal data as a result of typing errors, and if not, the corresponding data were removed. Data analyses were performed on SPSS statistical software 18.0 (IBM SPSS Inc., USA). A value of p < 0.05 was defined as statistical significance.

## Results

### Clinical Characteristics of the Study Participants

**Table 1** showed the clinical characteristics of the total population and the three subgroups based on the LDL-C/Apo B ratio tertiles. Along with the LDL-C/Apo B ratio ascending, lnOC, lnCTx and lnPINP levels gradually increased (all *p <* 0.05). BMI, proportion of peripheral artery disease, statins use, use of acarbose and TG level were the highest in T1, followed by T2 and T3, while TC and Apo B levels were the highest in T3, followed by T2 and T1 (all *p <* 0.05). Among the three subgroups, there were significant differences in percentage of hypertension, use of β-blocker and Apo A level (all *p <* 0.05). However, there were no differences in age, proportion of males, diabetic duration, systolic/diastolic blood pressure, use of other antidiabetic treatments and other antihypertensive treatments, HbA1c level, HOMA-IR_CP_ level, HDL-C level, LDL-C level, UACR level, eGFR level, proportion of diabetic kidney disease, PTH level, vitamin D level, lumbar spine and hip T-score, proportion of osteoporosis (all p > 0.05).

**Table 1:** Clinical characteristics of the study participants.

Variables	Total	T1	T2	T3	*p* value
LDL-C/Apo B ratio	2.68 (2.43-2.89)	<2.54	2.54-2.80	>2.80	
LDL-C/Apo B ratio (range)	0.78-4.00	0.78-2.53	2.53-2.81	2.83-4.00	
*n*	335	112	115	108	
Age (years)	58.37 ± 12.70	58.80 ± 12.46	57.55 ± 13.28	58.79 ± 12.39	0.920
Male, *n* (%)	178 (53.1)	60 (53.6)	65 (56.5)	53 (49.1)	0.534
Diabetic duration (years)	6.0 (2.0-10.0)	6.5 (2.0-10.0)	6.0 (1.0-10.0)	7.0 (2.0-10.0)	0.843
BMI (kg/m^2^)	25.54 ± 3.82	25.94 ± 4.46	25.77 ± 3.60	24.91 ± 3.30	0.027
Hypertension, *n* (%)	118 (35.2)	47 (42.0)	35 (30.4)	36 (33.3)	0.008
SBP (mmHg)	137.70 ± 19.69	136.56 ± 20.21	137.53 ± 18.45	139.06 ± 20.50	0.440
DBP (mmHg)	83.55 ± 11.33	83.08 ± 12.35	84.37 ± 11.02	83.18 ± 10.59	0.287
PAD, *n* (%)	164 (49.0)	65 (58.0)	58 (50.4)	41 (38.0)	0.011
Antidiabetic treatments					
Insulin treatment, *n* (%)	91 (27.1)	26 (23.2)	27 (23.5)	38 (35.2)	0.075
Metformin, *n* (%)	158 (47.2)	61 (54.5)	52 (45.2)	45 (41.7)	0.144
Acarbose, *n* (%)	23 (6.9)	15 (13.4)	5 (4.3)	3 (2.8)	0.003
Insulin-secretagogues, *n* (%)	106 (31.6)	36 (32.1)	42 (36.5)	28 (25.9)	0.233
Insulin-sensitizers, *n* (%)	34 (10.1)	11 (9.8)	12 (10.4)	11 (10.2)	0.988
DPP-4 inhibitors, *n* (%)	27 (8.1)	9 (8.0)	9 (7.8)	9 (8.3)	0.990
SGLT-2 inhibitors, *n* (%)	29 (8.7)	14 (12.5)	10 (8.7)	5 (4.6)	0.116
Antihypertensive treatments					
CCB, *n* (%)	84 (25.1)	36 (32.1)	26 (22.6)	22 (20.4)	0.099
ARB, *n* (%)	75 (22.4)	31 (27.7)	26 (22.6)	18 (16.7)	0.146
β-blockers, *n* (%)	23 (6.9)	14 (12.5)	4 (3.5)	5 (4.6)	0.014
Diuretics, *n* (%)	31 (9.3)	10 (8.9)	11 (9.6)	10 (9.3)	0.986
Statins medications, *n* (%)	22 (6.6)	18 (16.1)	3 (2.6)	1 (0.9)	<0.001
HbA1c (%)	9.27 ± 2.06	9.46 ± 2.15	9.19 ± 2.02	9.16 ± 2.06	0.701
HOMA-IR_CP_	0.45 (0.24-0.72)	0.53 (0.29-0.74)	0.44 (0.28-0.79)	0.42 (0.21-0.68)	0.386
UACR (mg/g)	16.25 (8.00-44.20)	16.20 (8.85-42.05)	17.10 (7.98-53.85)	15.90 (7.40-37.10)	0.384
eGFR (ml/min/1.73m^2^)	97.52 ± 28.02	96.74 ± 27.79	97.95 ± 28.70	97.83 ± 27.81	0.946
CKD, *n* (%)	45 (13.4)	21 (18.8)	13 (11.3)	11 (10.2)	0.126
TG (mmol/L)	1.66 (1.07-2.59)	2.09 (1.27-3.67)	1.64 (1.12-2.49)	1.28 (0.90-2.02)	<0.001
TC (mmol/L)	4.26 (3.72-5.02)	3.69 (2.98-4.38)	4.16 (3.80-4.78)	4.98 (4.37-5.46)	<0.001
HDL-C (mmol/L)	1.11 ± 0.26	1.01 ± 0.25	1.11 ± 0.24	1.23 ± 0.24	0.986
LDL-C (mmol/L)	2.76 ± 0.88	2.06 ± 0.71	2.85 ± 0.69	3.39 ± 0.67	0.914
Apo A (mmol/L)	1.10 (1.00-1.27)	1.04 (0.95-1.17)	1.09 (0.91-1.17)	1.08 (0.99-1.29)	<0.001
Apo B (mmol/L)	1.02 (0.87-1.18)	0.94 (0.74-1.07)	1.04 (0.91-1.17)	1.08 (0.99-1.29)	<0.001
lnOC	2.39 ± 0.47	2.27 ± 0.48	2.41 ± 0.42	2.48 ± 0.49	0.007
lnCTx	-0.88 ± 0.57	-0.97 ± 0.64	-0.85 ± 0.54	-0.81 ± 0.52	0.040
lnPINP	3.64 ± 0.47	3.54 ± 0.45	3.68 ± 0.42	3.70 ± 0.53	0.030
PTH (pg/mL)	34.95 (27.13-46.60)	33.55 (24.28-45.75)	37.80 (29.50-46.60)	34.10 (26.50-46.60)	0.105
Vitamin D (ng/mL)	16.42 ± 6.95	15.78 ± 6.90	16.22 ± 6.56	17.30 ± 7.39	0.364
Lumbar spine T-score	-1.35 (-2.28–0.225)	-1.85 (-2.63–0.75)	-1.60 (-2.00–0.80)	-1.10 (-2.40-0.00)	0.708
Hip T-score	-1.10 (-1.80–0.20)	-1.20 (-1.80–0.20)	-0.90 (-1.80-0.20)	-1.20 (-2.30–0.20)	0.439
OP, *n* (%)	111 (33.1)	38 (33.9)	32 (27.8)	41 (37.6)	0.458

Normally distributed values in the table are given as the mean ± SD, skewed distributed values are given as the median (25 and 75% interquartiles), and categorical variables are given as frequency (percentage).

LDL-C/Apo B ratio low-density lipoprotein cholesterol/apoprotein B ratio, BMI body mass index, SBP/DBP, systolic/diastolic blood pressure, PAD peripheral artery disease, Insulin-secretagogues insulin secretagogues, Insulin-sensitizers insulin sensitizing agents, DPP-4 inhibitors dipeptidyl peptidase-4 inhibitors, sodium-glucose co-transporter-2 inhibitors SGLT-2 inhibitors, CCB calcium channel blockers, ARB angiotensin receptor blockers, HbA1c glycosylated hemoglobin A1c, UACR urinary albumin-to-creatinine ratio, eGFR estimated glomerular filtration rate, CKD chronic kidney disease, TG triglyceride, TC total cholesterol, HDL-C high-density lipoprotein cholesterol, LDL-C low-density lipoprotein cholesterol, Apo A apolipoprotein A, Apo B apolipoprotein B, OC osteocalcin, CTx C-terminal telopeptide, PINP N-terminal propeptide of type-I procollagen, PTH parathyroid hormone, OP osteoporosis.

### Relationships Between the LDL-C/Apo B Ratio and BTMs

As illustrated in **Table 2**, the LDL-C/Apo B ratio was positively associated with lnOC, lnCTx and lnPINP levels (*r* = 0.244, 0.226 and 0.211, respectively, all *p* < 0.001). These significant and positive correlations persisted even when divided into male and female subgroups.

**Table 2:** Relationships between the LDL-C/Apo B ratio and BTMs.

Variables	Total		Male		Female	
*n*	335		178		157	
	*r*	*p* value	*r*	*p* value	*r*	*p* value
lnOC	0.244	<0.001	0.193	0.010	0.286	<0.001
lnCTx	0.226	<0.001	0.202	0.007	0.228	0.004
lnPINP	0.211	<0.001	0.152	0.043	0.251	0.002

r Pearson’s correlation coefficient.

### Multiple Linear Regression Models Displayed Independent Associations of the LDL-C/Apo B Ratio With BTMs Levels

In **Table 3**, the LDL-C/Apo B ratio was significantly and positively associated with lnOC level (β = 0.244, t = 4.582, p < 0.001, R^2^ = 0.059), lnCTx level (β = 0.226, t = 4.220, p < 0.001, R^2^ = 0.051) and lnPINP level (β = 0.211, t = 3.933, p < 0.001, R^2^ = 0.045). After adding the other clinical covariates in each model step by step, the R^2^ was gradually increased. In the model 1, after adjusting for age, sex, diabetic duration, BMI, systolic/diastolic blood pressure and smoking history, the LDL-C/Apo B ratio was significantly and positively associated with lnOC level (β = 0.193, t = 3.426, p < 0.01, R^2^ = 0.073), lnCTx level (β = 0.213, t = 3.885, p < 0.001, R^2^ = 0.127) and lnPINP level (β = 0.158, t = 2.860, p < 0.01, R^2^ = 0.109). Antidiabetic treatments, antihypertensive treatments and statins medications were then added as clinical covariates in the model 2, and the LDL-C/Apo B ratio was significantly and positively associated with lnOC level (β = 0.192, t = 3.238, p < 0.01, R^2^ = 0.107), lnCTx level (β = 0.219, t = 3.833, p < 0.001, R^2^ = 0.175) and lnPINP level (β = 0.155, t = 2.644, p < 0.01, R^2^ = 0.131). The fully adjusted model 3 further adjusted for HbA1c, HOMA-IR_CP_ and eGFR, the LDL-C/Apo B ratio was still significantly and positively associated with lnOC level (β = 0.199, t = 3.348, p < 0.01, R^2^ = 0.171), lnCTx level (β = 0.238, t = 4.084, p < 0.001, R^2^ = 0.202) and lnPINP level (β = 0.162, t = 2.741, p < 0.01, R^2^ = 0.172).

**Table 3:** Multiple linear regression models displayed independent associations of the LDL-C/Apo B ratio with BTMs levels.

Models	B (95% CI)	*β*	*t*	*p*	*R^2^* for model
lnOC					
Model 0	0.278 (0.158-0.397)	0.244	4.582	<0.001	0.059
Model 1	0.213 (0.091-0.335)	0.193	3.426	0.001	0.073
Model 2	0.212 (0.083-0.341)	0.192	3.238	0.001	0.107
Model 3	0.220 (0.091-0.350)	0.199	3.348	0.001	0.171
lnCTx					
Model 0	0.312 (0.167-0.457)	0.226	4.220	<0.001	0.051
Model 1	0.295 (0.146-0.445)	0.213	3.885	<0.001	0.127
Model 2	0.304 (0.148-0.460)	0.219	3.833	<0.001	0.175
Model 3	0.333 (0.172-0.493)	0.238	4.084	<0.001	0.202
lnPINP					
Model 0	0.241 (0.121-0.362)	0.211	3.933	<0.001	0.045
Model 1	0.178 (0.055-0.300)	0.158	2.860	0.005	0.109
Model 2	0.174 (0.045-0.304)	0.155	2.644	0.009	0.131
Model 3	0.184 (0.052-0.316)	0.162	2.741	0.007	0.172

Model 0, unadjusted model.

Model 1, adjusted for age, sex, diabetic duration, BMI, SBP, DBP, smoking history.

Model 2, additionally adjusted for antidiabetic treatments, antihypertensive treatments, statins medications.

Model 3, additionally adjusted for HbA1c, HOMA-IR_CP_, eGFR.

## Discussion

In the present study, we investigated the association between the LDL-C/Apo B ratio with BTMs in patients with T2D. The main findings are as follows: first, with the increase of the LDL-C/Apo B ratio tertile, serum OC, CTx and PINP levels gradually increased; second, the LDL-C/Apo B ratio was significantly and positively related to serum OC, CTx and PINP levels; third, after adjusting for sex, age, diabetic duration and other clinical factors *via* multiple linear regression analysis, the LDL-C/Apo B ratio was still significantly and positively associated with serum OC, CTx and PINP level. Collectively, a decreased LDL-C/Apo B ratio is independently associated with low bone turnover in patients with T2D.

Existing guidelines recommend detecting BMD (T-score) *via* DXA for the initial diagnosis of osteoporosis, and osteoporosis is diagnosed based on a T-score lower than or equal to -2.5 at the spine or hip (19). However, the Rotterdam study showed an increased risk of fracture despite higher BMD in patients with T2D or impaired glucose tolerance (IGT) compared with those with normal glucose metabolism (20). A meta-analysis got similar results that with BMDs in patients with T2D were 4-5% higher than in healthy population (21). These results suggested that BMD should not be the only concern for bone health in patients with T2D. Manavalan et al. performed bone biopsies in patients with T2D and controls, and found significant reduced bone formation rate, osteoid surface, and osteoblast surface in T2D patients, suggesting the importance of bone turnover (22). Most studies demonstrated that bone turnover in patients with T2D was decreased (5, 23, 24). Hence, the present study aimed to explore the relationship between the LDL-C/Apo B ratio and BTMs in patients with T2D.

Risk factors for low bone turnover in patients with T2D include glucose control, use of hypoglycemic drugs, hyperlipidemia, advanced glycation end products (AGEs), bone microvascular disease and so on (25). Poor glycemic control is the initiating factor of the onset and progression of diabetic chronic complications, and a cross-sectional study of men with T2D revealed that a HbA1c greater than 7% was closely associated with decreased bone turnover (26). Reactive oxygen species (ROS) is an important mediator of diabetic chronic complications induced by hyperglycemia, hyperlipemia and AGEs (27). ROS can directly inhibit Wnt/β-catenin signaling pathway, which is critical for bone formation (28). ROS is also a key signaling molecule mediating inflammation, which induces the expression of Dickkopf-related protein 1 (DKK1) (29). Subsequently, DKK1 can bind to lipoprotein receptor-related protein 6 (LRP6) to form a complex contributing to the internalization of LRP6. LRP6, a member of the LDLR gene family, is a co-receptor of Wnt, and the internalization of LRP6 can lead to the inhibition of Wnt/β-catenin signaling pathway (30). In addition, in hyperlipidemia rat model hyperlipidemia could inhibit dishevelled-2 expression and its phosphorylation, thus inhibiting Wnt/β-catenin signaling pathway (31). Consistently, the present study found that a low LDL-C/Apo B ratio was an independent contributor to low bone turnover in patients with T2D.

LDL-C is an important risk factor for atherosclerosis, and a study revealed that serum LDL-C was an independent risk factor for fragility fractures in postmenopausal women (32). Compared with other LDL-Cs, sd-LDL-C has delayed catabolism and increased oxidation susceptibility (33). In high-fat fed rats, oxidized LDL could inhibit bone formation by blocking osteoblast progenitor cells differentiation and inhibiting OC expression in marrow (34). Similarly, in our study the LDL-C/Apo B ratio was positively associated with serum OC and PINP levels. Additionally, we also observed a positive relationship between the LDL-C/Apo B ratio and CTx level. However, oxidative lipids could promote the differentiation of marrow preosteoclasts *in vitro*, so oxidized lipids might be positively related to serum CTx level (35). This discrepancy may be explained by other mechanisms of low bone turnover in T2D.

Bone microvascular disease is also a vital pathogenesis of decreased bone turnover in patients with T2D (13). As well as peripheral vessels, a histological study showed that intraosseous arterioles also occurred atherosclerosis plagues (36). Legs without atherosclerotic plaques had higher BMD than those with atherosclerotic plaque (37). In addition, the accumulation of oxidized lipoprotein particles was observed in the subcutaneous space surrounding blood vessels (38). In the present study, proportion of peripheral artery disease (PAD) increased from the first tertile to the third tertile of the LDL-C/Apo B ratio. These results highlighted the role of lipids in affecting bone turnover by inducing bone microvascular injury.

In this study, the LDL-C/Apo B ratio was significantly and positively correlated with BTMs levels even after adjusting for other covariates. In line with these results, a review suggested that dyslipidemia might aggravate atherosclerosis independently of serum LDL-C levels, possibly due to the presence of sd-LDL-C (39). Worse, uses of statin did not significantly reduce serum sd-LDL-C levels in patients with acute ischemic stroke (40). Similarly, the present study also observed that proportion of statin use was highest in the first tertile of the LDL-C/Apo B ratio. Therefore, adequate attention should be paid to the LDL-C/Apo B ratio in patients with T2D.

Although the present study observed an independent positive correlation between the LDL-C/Apo B ratio and BTMs, we failed to observe a correlation between the LDL-C/Apo B ratio and T-score. This was similar to other studies (26, 41), which might ascribe to the fact that BMD or T-score could not fully reflect bone alternations in T2D.

Several limitations of this study should be pointed out. First, the causal relationship between the LDL-C/Apo B ratio and bone turnover could not be concluded based on the present study, which was an observational cross-sectional study. Longitudinal and intervention studies are needed to address the limitation. Second, although we intended to present the trend of BTMs among the LDL-C/Apo B ratio tertiles in **Table 1**, grouping by the tertiles of LDL-C/Apo B ratio had no biological significance. In future studies, the association between the LDL-C/Apo B ratios and sd-LDL-C levels needs to be evaluated, and then grouping may be more meaningful than grouping directly by the tertiles of this ratio. Third, the LDL-C/Apo B ratio is a substitute index rather than the gold standard for evaluating sd-LDL-C, but the relationship between LDL-C/Apo B ratio and sd-LDL-C has been fully verified by multiple clinical studies. Fourth, high-resolution peripheral quantitative computed tomography (HRpQCT) may reflect bone microarchitectural properties better than DXA, so HRpQCT should be carried out simultaneously in future studies. Fifth, the generalization of this study was limited by the fact that all subjects enrolled in this study were Chinese.

In a word, the LDL-C/Apo B ratio was significantly and positively associated with BTMs in patients with T2D. In clinical practice, more attention should be paid to the patients with T2D whose LDL-C/Apo B ratio is relatively low for the purpose of maintaining bone health. This study is a hypothesis generating study, and we hypothesis that a predominance of sd-LDL-C in LDL-Cs may be an important risk factor for low bone turnover in patients with T2D by inducing oxidative stress and artery trauma. This hypothesis requires further follow-up and intervention studies to evaluate the effects of sd-LDL-C on bone turnover in patients with T2D, and to explore the mechanisms of sd-LDL-C inducing low bone turnover in animal and cell experiments.

## Data Availability Statement

The raw data supporting the conclusions of this article will be made available by the authors, without undue reservation.

## Ethics Statement

The studies involving human participants were reviewed and approved by the medical research ethics committee of Second Affiliated Hospital of Nantong University. The patients/participants provided their written informed consent to participate in this study.

## Author Contributions

Cf-L and W-sL participated in the design of the study, data collection, analysis of the data, and drafting of the manuscript. X-qW and J-bS conceived of the study, participated in its design and revised the manuscript. X-qG, H-yH, and L-yH participated in data collection. All authors contributed to the article and approved the submitted version.

## Funding

The study was supported by the Medical Research Project of Health Commission of Nantong (MB2020012) and the Science and Technology Support Program of Nantong (HS2020005).

## Conflict of Interest

The authors declare that the research was conducted in the absence of any commercial or financial relationships that could be construed as a potential conflict of interest.

## Publisher’s Note

All claims expressed in this article are solely those of the authors and do not necessarily represent those of their affiliated organizations, or those of the publisher, the editors and the reviewers. Any product that may be evaluated in this article, or claim that may be made by its manufacturer, is not guaranteed or endorsed by the publisher.
